# Coaxial Electrospun Nanofibrous Membranes for Enhanced Water Recovery by Direct Contact Membrane Distillation

**DOI:** 10.3390/polym14245350

**Published:** 2022-12-07

**Authors:** Vivekanandan Sangeetha, Noel Jacob Kaleekkal, Saravanamuthu Vigneswaran

**Affiliations:** 1 Membrane Separation Group, Department of Chemical Engineering, National Institute of Technology Calicut, Kozhikode 673601, Kerala, India; 2 Centre for Technology in Water and Wastewater, School of Civil and Environmental Engineering, University of Technology Sydney, Sydney, NSW 2007, Australia; 3 Faculty of Sciences & Technology (RealTek), Norwegian University of Life Sciences, P.O. Box 5003, NO-1432 Ås, Norway

**Keywords:** coaxial, electrospun membrane, omniphobic, hypersaline, surfactants, cationic, anionic, non-ionic

## Abstract

Membrane distillation (MD) is an emerging technology for water recovery from hypersaline wastewater. Membrane scaling and wetting are the drawbacks that prevent the widespread implementation of the MD process. In this study, coaxially electrospun polyvinylidene fluoride-co-hexafluoropropylene (PVDF-co-HFP) nanofibrous membranes were fabricated with re-entrant architecture and enhanced hydrophobicity/omniphobicity. The multiscale roughness was constructed by incorporating Al_2_O_3_ nanoparticles and 1H, 1H, 2H, 2H Perfluorodecyltriethoxysilane in the sheath solution. High resolution transmission electron microscopy (HR-TEM) could confirm the formation of the core-sheath nanofibrous membranes, which exhibited a water contact angle of ~142.5° and enhanced surface roughness. The membrane displayed a stable vapor flux of 12 L.m^−2^.h^−1^ (LMH) for a 7.0 wt.% NaCl feed solution and no loss in permeate quality or quantity. Long-term water recovery from 10.5 wt.% NaCl feed solution was determined to be 8–10 LMH with >99.9% NaCl rejection for up to 5 cycles of operation (60 h). The membranes exhibited excellent resistance to wetting even above the critical micelle concentration (CMC) for surfactants in the order sodium dodecyl sulphate (SDS) (16 mM) > cetyltrimethylammonium bromide (CTAB) (1.5 mM) > Tween 80 (0.10 mM). The presence of salts further deteriorated membrane performance for SDS (12 mM) and Tween-80 (0.05 mM). These coaxial electrospun nanofibrous membranes are robust and can be explored for long-term applications.

## 1. Introduction

The acute freshwater shortage is one of the most challenging crises that demand prompt attention. Desalination employing reverse osmosis (RO) is widely adopted to recover water and meet the increasing demand [1]. RO is a pressure-driven membrane-based process that now accounts for >60% of desalination plants worldwide [2]. However, the RO process rejects approximately 40% of highly concentrated brine which is quite detrimental to the ecosystem [3]. Apart from RO reject, the common sources of hypersaline wastewater originate from produced water (oil or shale gas), industrial effluents (tanning, mining, etc.), and thermoelectric power plants [4]. Water recovery from these hypersaline wastewaters can augment freshwater resources, reduce the total volume of dewatered brine handled, and help meet the government requirements of zero-liquid discharge [4,5].

Membrane distillation (MD) is an emerging technology that can handle hypersaline feed streams and recover freshwater. The direct contact membrane distillation (DCMD) configuration employs a hydrophobic/superhydrophobic microporous membrane that physically separates the hot feed and the cooled distillate (permeate). The water vapours or volatile components (from the feed) are transported across this membrane due to the transmembrane vapour pressure gradient arising from the temperature difference between the two streams (hot feed and cold permeate) [6,7]. The advantages of the DCMD process include (i) the ability to handle high salt concentration (RO cannot handle >7 wt.% NaCl), (ii) theoretically 100% rejection of non-volatiles (inorganic salts), (iii) operation at lower temperatures and at atmospheric pressure, (iv) utilization of waste heat or low-grade energy to heat the feed, (v) integration with renewable energy sources and (vi) provides an option for a decentralized desalination system. [8,9]. The widespread implementation of this technology is still limited owing to some demerits such as membrane wetting, fouling and scaling [10]. Scaling or inorganic fouling is severe while treating hypersaline wastewater and depends on the temperature and solute concentration at the liquid–vapor interface [11]. Concentration polarization (CP) occurs due to the accumulation of the inorganic ions retained near the feed-membrane interphase during the MD process, which impairs the membrane performance (lower vapor flux and higher permeate conductivity). The salt barrier (external CP) leads to further deposition into the membrane pores and blocks the pores or wets the membrane (internal CP) [12]. Further, the salt barrier induces heat transfer resistance, aggravating the temperature polarization (TP) phenomenon and lowering the driving force [13].

In addition to the high concentration of inorganic salts, another class of compounds—surfactants—are commonly found in wastewaters from textile, food processing, paint and polymer manufacturing units, automobile industries, mining, oil refineries, paper and pulp industries, pharmaceuticals manufacturing units, petrochemical refineries, laundry wastewater and domestic wastewater [14,15,16]. Surfactants in domestic wastewater can range from 1 to 10 mg/L, while wastewater from surfactant manufacturing industries can reach up to 300 mg/L [17]. Surfactants are amphipathic molecules comprising a hydrophilic (polar charged or uncharged head group) and a hydrophobic (non-polar hydrocarbon tail). Surfactants are classified based on the charge type on the hydrophilic groups as anionic (negatively charged functional group), cationic (positively charged functional group), non-ionic (non-ionized hydrophilic group), semi-polar and amphoteric (charge changes as a function of pH) [18]. Surfactants are widely used to reduce the interfacial tension between liquid-liquid/solid molecules. Surfactants in wastewater pose a significant challenge to DCMD as they reduce the interfacial tension and subsequently reduce the membrane’s liquid entry pressure (LEP), leading to loss of permeate quality (membrane wetting phenomena) [19]. The adsorption of the surfactant onto the membrane pores can also lead to membrane wetting as it forms hydrophilic channels, which can aid in the transport of feed water which deteriorates permeate quality [20].

Electrospun nanofibrous membranes (ENFMs) are gaining interest as potential candidates for MD membranes due to their tunable hydrophobicity, flexible pore structure, low tortuosity and high porosity [21]. All interaction between the feed solution–membrane or solute–membrane interactions, such as adsorption and electrostatic interaction (hydrophobic–hydrophobic), occurs on the top layer of the membrane facing the feed solution. Several membrane modifications have been implemented to improve the anti-scaling or anti-wetting ability of the membranes, of which the incorporation of suitable nanoparticles is the most promising [22]. The hierarchical surface architecture of the membrane featuring the re-entrant structure impedes the conversion of the steady Cassie–Baxter (CB) state to the Wenzel state, which prevents membrane wetting [23]. Enhancing membrane hydrophobicity is a versatile approach to modify membranes to hamper pore intrusion by the feed (liquid) [8]. However, amphiphilic compounds such as surfactants (surface active agents) induce and promote membrane pore wetting leading to the failure of the MD process [20]. Therefore, it is imperative to fabricate a robust superamphiphobic/superomniphobic membrane with both low surface free energy and a re-entrant architecture, and this synergistic effect can make the liquid–solid–vapor interfaces in the metastable Cassie–Baxter state [24].

Various membrane fabrication approaches have been explored to prepare robust membranes with excellent anti-scaling and anti-wetting properties. For instance, a dual-layered electrospun nanofibrous membrane was fabricated, featuring surface fluorinated mesoporous silica nanoparticles on the top layer [25]. The membrane exhibited re-entrant morphology with an excellent water contact angle (143°), stable flux, salts and organic compounds rejection (>99%). Electrospraying is yet another approach to construct the re-entrant architecture on the membrane surface uniformly. A dual-layered electrosprayed-electrospun polyvinylidene fluoride-co-hexafluoropropylene (PVDF-co-HFP) membrane surface grafted with fluoro alkyl silane (FAS) functionalized zinc oxide (ZnO) featuring re-entrant morphologies demonstrated contact angles with water, oil and ethanol as 161°, 131.5° and 131.29° [23]. The membrane demonstrated a stable flux and salt rejection >99.9% during the 80 h of operation with 0.1 mM and 1 M NaCl feed solution. An electrospun and electrospraying technique was adopted to fabricate PVDF-co-HFP fibers with electrosprayed PS (polystyrene) beads membrane for direct contact membrane distillation [26]. The fabricated membrane featured randomly dispersed polystyrene beads forming distinct protrusions and caves on the surface of the membrane (primary hierarchical structure). Further, the nanopores formation constructed a secondary hierarchical structure. The membrane demonstrated a water contact angle of 157.6° attributed to the combined effect of primary and secondary hierarchical roughness, high salt rejection rate (~99%), stable flux, and tolerance for sodium dodecyl sulphate (SDS) concentrations of 0.2 mM.

The role of different surfactants such as SDS, cetyltrimethylammonium bromide (CTAB) and Tween-20 in wetting a commercial PVDF membrane (0.45 µm) was explored in detail [7]. The results inferred that the membrane wetting was instantaneous in the case of CTAB, followed by Tween 20 and SDS. However, the hydrophobicity of the membranes could be recovered for CTAB and SDS but not for Tween-20. In another study, it was demonstrated that the anionic surfactant with greater hydrophobicity deteriorates the MD performance, which is further aggravated by the presence of salt in the feed. Modification using surface-energy-reducing agents and silica nanoparticles rendered the membrane surfaces omniphobic and proved helpful in preventing membrane wetting or scaling during water recovery using DCMD from unconventional oil and gas wastewater. However, for long-term water recovery from the feed containing surfactants used in hydraulic fracturing fluids, the omniphobic membranes also succumb to membrane scaling and wetting. Hence, pre-treating the feed is a better strategy to mitigate the deterioration of permeate quality [27].

Coaxial electrospinning is a facile, single-step strategy to fabricate robust amphiphobic coaxial electrospun nanofibrous membranes (CENMs) with three-dimensional hierarchical roughness. Dual-layered fibers with tunable core or sheath properties make this strategy versatile for producing superior membranes for MD [28]. The nanoparticles of interest and FAS can be incorporated into the sheath solution and electrospun coaxially. This facilitates the formation of a fibrous network with multiscale re-entrant structures (enhanced hydrophobicity) and highly fluorinated surfaces (low surface energy). This method requires ultra-low concentrations of nanoparticles and FAS.

In the present study, coaxial fibers were prepared using PVDF-co-HFP for the core solution and Al_2_O_3_ nanoparticles/1H, 1H, 2H, 2H Perfluorodecyltriethoxysilane (FAS)/PVDF-co-HFP for the sheath. Alumina was incorporated to improve fiber roughness, and FAS was incorporated to reduce the surface free energy of the membrane [29]. The fabricated CENMs demonstrated good stability in recovering water from hypersaline wastewater. The role of various surfactants (cationic, anionic and non-ionic) and the influence of salts in the wetting behavior were elucidated in detail. This one-step fabrication of hydrophobic/amphiphobic membranes was determined to have great potential to be explored for long-term water recovery studies.

## 2. Materials and Methods

### 2.1. Materials

Polyvinylidene fluoride-co-hexafluoropropylene (PVDF-co-HFP, average Mw~400,000), 1H, 1H, 2H, 2H perfluorodecyltriethoxysilane (FAS, 97%), hexadecane (99%) was procured from Sigma-Aldrich, St. Louis, MO, USA. N, N dimethylformamide (DMF, >99.5%), acetone (>99.5%), isopropyl alcohol (IPA, >99.5%), sodium dodecyl sulphate (SDS, >99.0%) cetyltrimethylammonium bromide (CTAB, >99.0%), Tween-80 (≥98%) and alumina nanopowder—Alpha (20–30 nm, >99.9%) were obtained from Sisco Research Laboratories Pvt. Ltd., Mumbai, India. Analytical reagent grade solvents such as ethanol and methanol (>99.5%) and sodium chloride (NaCl, ≥99.0%), hydrochloric acid 35% (HCl, >99.5%) and sodium hydroxide (NaOH, 97%) were obtained from Merck, Mumbai India and used without further purification. Distilled water (<2 µS/cm) was used throughout the study.

### 2.2. Preparation of Coaxial Electrospun Nanofibrous Membranes

**Figure 1:**
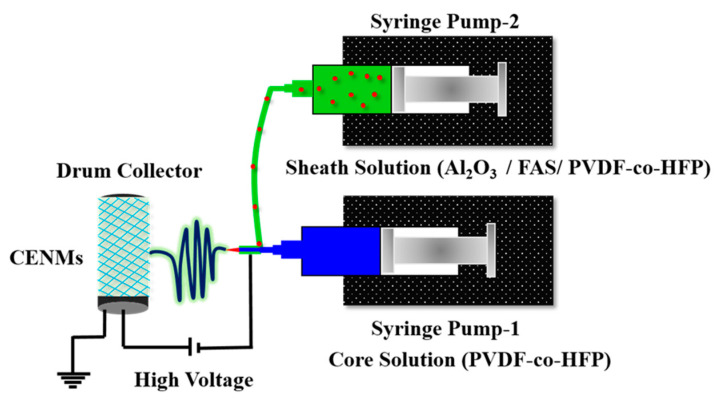
Illustration of coaxial electrospinning setup and CENM fabrication.

The core and sheath electrospun nanofibrous membranes were prepared per the composition in Table 1. The polymer dope solutions were prepared by dissolving PVDF-co-HFP in the solvent mixture by magnetic stirring for 4 h at room temperature. Alumina nanoparticles were dispersed homogeneously in the solvent mixture using an ultrasonic bath (GT Sonic, An-SS-3L, Guangdong, China) for 20 min before dissolving the polymer. A nanofiber electrospinning unit (ESpin Nanotech, SUPER ES-2, Kanpur, India) equipped with a rotating drum collector and dual pump syringe system controlled using proprietary software was used to fabricate the membranes. The optimized electrospinning conditions for the core and sheath membranes are as follows: (a) tip-to-collector distance—15 cm, (b) drum speed—2000 rpm, (c) flow rate—1.7 mL/h (Core) and 1 mL/h (Sheath), (d) voltage—18 kV, (e) temperature—35 °C, (f) relative humidity—80% and (g) needle—21 G (Core) and 18 G (Sheath). The core and sheath solutions were loaded into 10 mL syringes each and were connected to a coaxial spinneret. Upon application of high voltage, core-sheath nanofibers were formed and collected on the collecting drum (Figure 1). The membranes were hot pressed at 90 °C post fabrication, washed with ethanol and dried at 60 °C for 12 h before their application in DCMD.

### 2.3. Characterization of Nanoparticles and Membranes

X-ray diffraction patterns of Al_2_O_3_ nanoparticles were obtained using X-Ray Diffractometer (Rigaku, Miniflex 600, Tokyo, Japan) with Cu Kα radiation (λ = 1.5406 Å). The chemical bonds were identified by the attenuated total reflectance (ATR) technique using Fourier transform infrared spectroscopy (Jasco, 4700, Tokyo, Japan) in the range of 400–4000 cm^−1^ (transmission mode). The surface morphology was examined using scanning electron microscope (Jeol, 6390LA, Mitaka, Japan) and high-resolution transmission electron microscope (HR-TEM, Jeol, JEM2100, Tokyo, Japan) equipped with 200 kV LaB6 electron gun with a point resolution of 0.23 nm and lattice resolution of 0.14 nm. The elemental composition was determined using energy dispersive X-Ray analysis (OXFORD, XMX N, Abingdon, UK) with a resolution of 136 eV and detector area of 30 mm^2^ respectively. The porosity and surface area of Al_2_O_3_ nanoparticles were measured using the BET apparatus (MicrotracBEL Corporation, BELSORP-max, Osaka, Japan). The contact angle for the membranes was measured using a contact angle goniometer (Kyowa Co., DMs-401, Kyowa, Japan) in sessile drop mode. Five individual measurements at different positions on the membrane were used to compute the average contact angle. The surface tension of the liquids was measured using a surface tension analyzer (SEO, Gyeonggi-do, Republic of Korea) and was taken as the average of 3 samples.

### 2.4. Evaluation of CENMs for Direct Contact Membrane Distillation

The experimental setup and procedure were similar to our previous work [25]. The composition of the feed solution and properties of the surfactants are given in Table 2 and Table S1, respectively.

## 3. Results

### 3.1. Characterization of Alumina Nanoparticles

**Figure 2:**
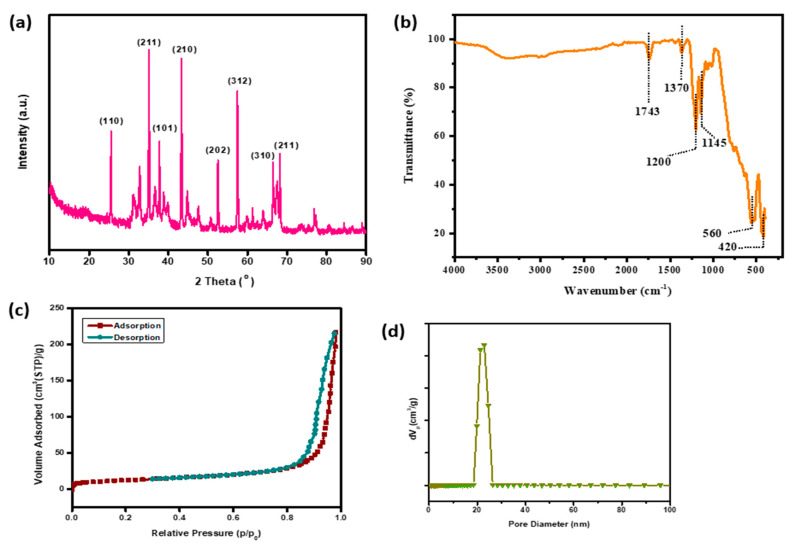
(**a**) XRD (**b**) FT-IR (**c**) BET (**d**) Pore size distribution of Alumina Nanoparticles.

**Figure 3:**
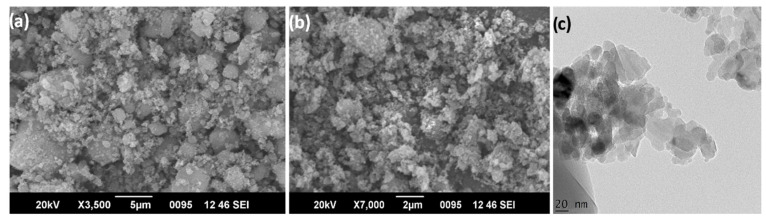
(**a**,**b**) FE-SEM images and (**c**) HR-TEM images of Alumina Nanoparticles.

α-Alumina is known to be the most stable phase of alumina [30]. The XRD analysis (Figure 2a) of the unmodified/hydrophilic alumina displayed the characteristic diffraction peak with the planes (110), (211), (101), (210), (202), (312), (310) and (211) denoting the rhombohedral crystalline structure of α-Alumina [31]. The FTIR spectra (Figure 2b) of Alumina exhibited the characteristic peaks at 560 cm^−1^ corresponding to the stretching vibration of Al-O band [32]. The BET and BJH analysis of the N_2_ adsorption-desorption curves (Figure 2c) yielded a specific surface area of 43.826 m^2^.g^−1^ with mean pore diameters of 30.55 nm and pore volumes of 10.069 cm^3^ (STP) g^−1^. The surface morphology of alumina nanoparticles was examined through FE-SEM (field emission-scanning electron microscopy) and HR-TEM images. The FE-SEM images display the aggregated nano spherical morphology of crystalline alumina with greater uniformity in size distribution (Figure 3a,b). HR-TEM imaging analysis further confirmed the crystalline nature of the Al_2_O_3_ (Figure 3c).

### 3.2. Characterization of CENMs

**Figure 4:**
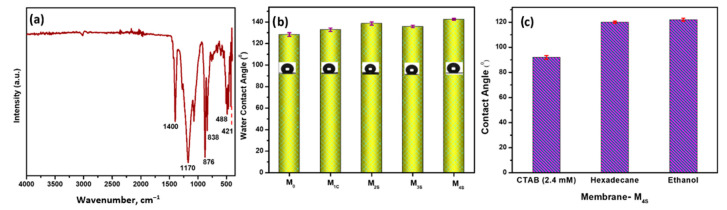
(**a**) FTIR, (**b**) WCA (optical images on the bar graph) and (**c**) CA with CTAB (2.4 mM), Hexadecane and Ethanol of Membrane M_4S_.

The FT-IR (Figure 4a) of the prepared membranes depicted characteristic peaks at 1400 cm^−1^, 1170 cm^−1^, 1070 cm^−1^, 839 cm^−1^ and 876 cm^−1^ corresponding to the antisymmetric stretching of -CF, the -CF_2_ stretching, the α phase of PVDF-co-HFP, the β phase of PVDF-HFP and the -CF stretching, respectively [23]. A characteristic peak at 470 cm^−1^ represents the bending vibration of Al-O present in the nanomaterial [33]. The water contact angle of the CENMs is presented in Figure 4b. The base membrane M_0_ exhibited the lowest water contact angle (WCA) of 128.3 ± 1.7° which could be attributed to the presence of hydrophilic alumina nanoparticles on the sheath of the CENM. The FAS modification by surface coating or incorporation in the sheath improved the WCA of the CENMs (>130°). M_2S_ demonstrated a higher water contact angle (138.6 ± 1.5°) which reveals the uniform functionalization of FAS on the fibrous network throughout the entire membrane. The addition of nanoparticles influences the surface roughness of the fibers and thereby enhances hydrophobicity [34]. When the hydrophilic alumina content was increased (0.2%), the WCA was slightly reduced. However, increasing the concentration of FAS in the sheath demonstrated an increase in the WCA for M_4S_. These WCA could be attributed to the hierarchical re-entrant morphology produced by the microscale CENMs, the nano-scale roughness produced by the incorporation on α -Al_2_O_3_, and the presence of the low-surface-energy FAS in the sheath [35].

Moreover, the amphiphobic character of CENM-M_4S_ could be confirmed by poor wetting of the membrane surface determined by the high contact angle measurements of various solutions/solvents. The contact angles of 2.4 mM CTAB solution (wetting concentration), hexadecane (non-polar oil) and ethanol (absolute) were determined to be 92.54 ± 2.4° 119.6 ± 1.8° and 121.6 ± 2.6°, respectively, indicating the anti-wetting ability of the membrane (Figure 4c) [36].

**Figure 5:**
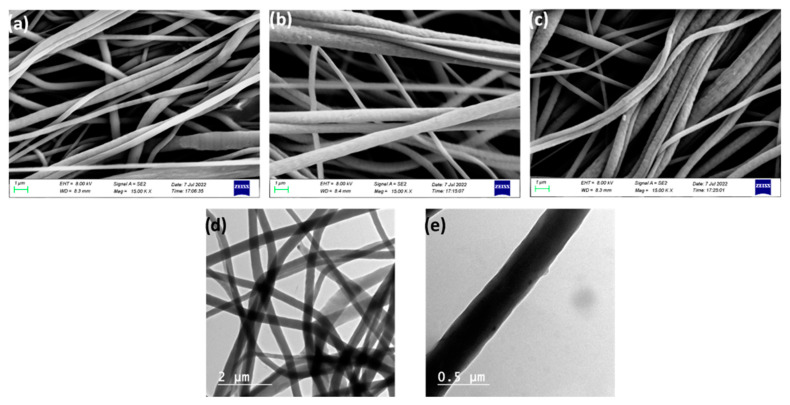
Surface morphology of prepared membranes. SEM image of (**a**) M_2S_, (**b**) M_3S_, (**c**) M_4S_, (**d**,**e**) HR-TEM image of M_4S_.

Smooth bead-free nanofibers are general formed by electrospinning polymers such as PVDF-co-HFP (not shown). Incorporating the Al_2_O_3_/FAS in the sheath layer produced a micro-nano hierarchical roughness on the fiber structure, which could enhance the anti-wetting ability of the membranes in good agreement with previous reports [28]. The SEM images indicate that the inner and outer solution flow rates were optimum, the CENMs produced were homogenous, and that a uniform Al_2_O_3_ dispersion could be achieved (Figure 5a–c). The increase in Al_2_O_3_ concentration leads to an increase in the average fiber diameter.

**Figure 6:**
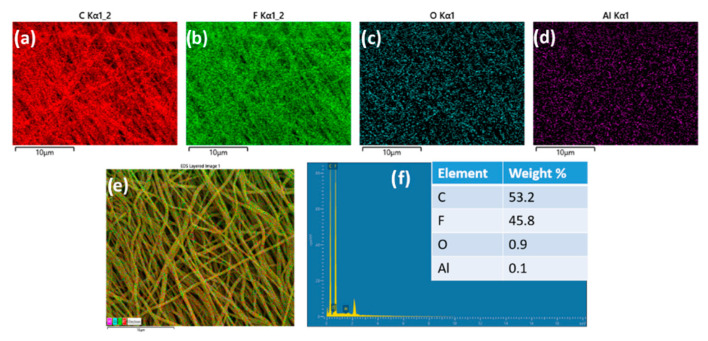
Color Mapping of M_4S_ (**a**) C, (**b**) F, (**c**) O and (**d**) Al (**e**) distribution of elements in the fibrous network and (**f**) elemental mapping of M_4S_ (inset: elemental composition).

The high-resolution TEM images (Figure 5d,e) revealed the successful formation of core and sheath electrospun nanofibers. The same polymer in both core and sheath solutions could lead to partial miscibility of the inner and other layers, as observed [37]. The elemental composition of the membrane using EDAX mapping further confirms the successful incorporation of Al_2_O_3_ on the fiber surface (Figure 6 and Table S2).

### 3.3. DCMD Performance of CENMs—Water Recovery from Hypersaline Wastewater

**Figure 7:**
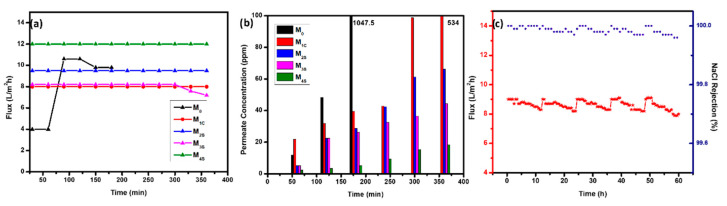
Performance of prepared membranes with 7.0% NaCL feed solution (**a**) Flux (**b**) Permeate Concentration. (**c**) Flux and NaCl rejection performance of membrane M4S with 10.5% NaCl feed solution.

The fabricated CENMs were evaluated in DCMD for water recovery from a synthetic hypersaline feed solution of 7 wt.% NaCl for 6 h (Figure 7a,b). The M_0_ membrane, without any FAS modification, displayed the lowest vapor permeate flux (4 LMH) during the initial 60 min of operation. The complete wetting of the membrane pores could be confirmed by the drastic increase in the vapor permeation rate and significant loss in permeate quality (NaCl rejection ≃ 98%) [10]. The increased membrane hydrophobicity by FAS (surface coating or introduction in the sheath solution) improved the vapor permeate flux. However, the M_1C_ also experienced partial pore wetting, confirmed by the continuous increase in the permeate salt concentration. Within 180 min, the salt concentration in the permeate reached 1047.5 ppm with no loss in the vapor permeation flux.

The introduction of FAS in the sheath ensured the complete coating of the low-surface-energy silane on the nanofibers. Further, the surface roughness became more prominent on increasing the concentration of both Al_2_O_3_ NPs and FAS, which improved the vapor transport rate even though a slight decline in membrane porosity was observed. The enhanced WCA and micro-nano hierarchical structures could hinder the heterogenous nucleation and the precipitation of salts from hypersaline feeds [38]. The membrane M_2S_ and M_3S_ also demonstrated a stable flux and salt rejection; however, the membrane M_3S_ showed a slight decline in the overall performance due to a relatively higher concentration of hydrophilic alumina nanoparticles (WCA—135.84 ± 1.1). The M_4S_ outperformed all the other membranes and displayed a stable permeate vapor flux and high rejection of NaCl (<20 ppm) and the results were reproducible.

The M_4S_ displayed the most outstanding vapor permeate flux and salt rejection; hence was challenged with an even higher concentration (10.5 wt.%) of NaCl feed solution (Figure 7c). The DCMD operation was carried out for 60 h with five operating cycles of 12 h per day. The membranes were flushed with DI water for 30 min and dried before each cycle. Throughout the run, the feed concentration was maintained at 10.5 wt.%. As expected, increasing feed concentration decreases vapor permeate flux due to lower vapor pressure that decreases driving force, which alters the transmembrane mass transport [39].

The M_4S_ demonstrated a consistent flux of 8–9 LMH for every cycle, and the salt rejection never fell below 99.97%. DI water wash could almost completely recover the initial water flux for up to five cycles of operation (Figure 5). In each cycle, the decrease in permeate vapor flux is due to the reduction in the feed water activity (increase in solution concentration), and partial loss in membrane hydrophobicity due to the reversible scaling (salt deposition) [40]. However, no loss in permeate quality could be observed, indicating that complete pore wetting did not occur. The unique membrane architecture of the CENMs and their hydrophobic nature could prevent membrane wetting for prolonged use [41].

### 3.4. DCMD Performance of CENMs—Effect of Surfactants

The presence of any surfactant lowers DCMD performance as they reduce the surface tension of the feed, leading to a lowering of the interface free energy. Further, the adsorption of these hydrophilic surfactants on the membrane surface and pores reduces the membrane hydrophobicity. These two factors can lead to the wetting of the membrane leading to a loss of permeate quality [42].

In this study, the wetting resistance of the M_4S_ was evaluated for different types of surfactants in the feed, namely—CTAB (cationic), SDS (anionic) and Tween 80 (non-ionic). The concentrations of individual surfactants were continuously increased (from a value lower than CMC to a concentration when instantaneous wetting occurs) at equal intervals. The presence of salt (3.5 wt.% NaCl) further lowered the surface tension of the feed and altered the surfactant–membrane and surfactant–surfactant interactions. This is also confirmed by the decrease in WCA measured (time dependent) as given in Table S3. For instance, the CMC value for CTAB is 0.9 mM. The initial CTAB concentration in the feed solution was 0.3 mM and gradually increased by 0.3 mM every 60 min until the complete wetting of the membrane. Similarly, the influence of the presence of salt (3.5% NaCl) along with the surfactants was also evaluated. The properties of the surfactants are given in Table S1.

**Figure 8:**
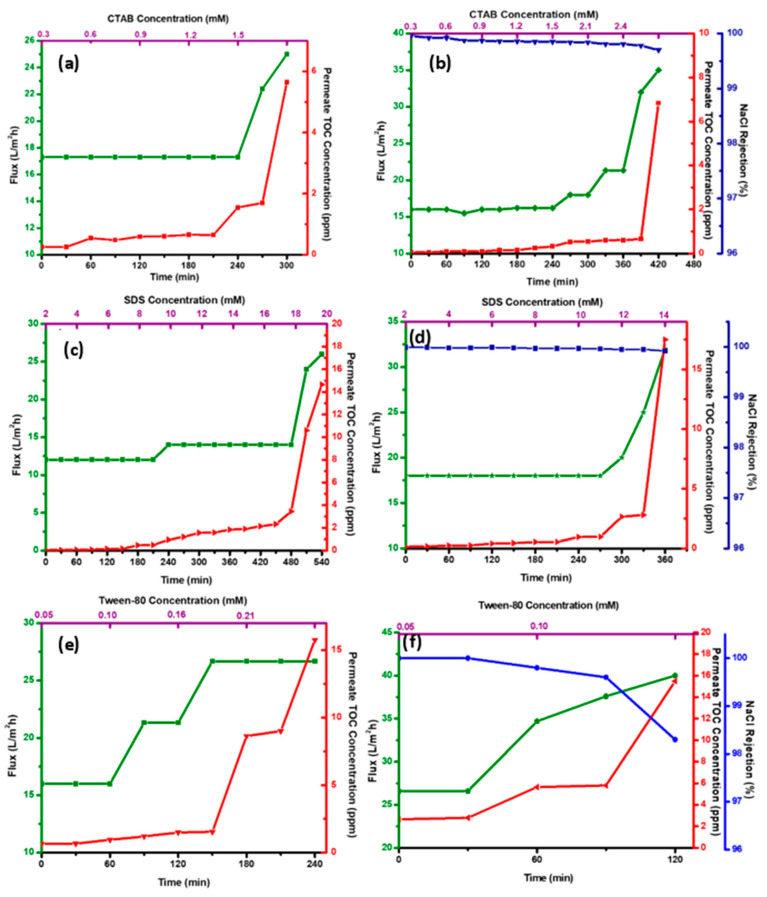
(**a**,**c**,**e**) Flux and Permeate concentration with CTAB, SDS and Tween 80, respectively. (**b**,**d**,**f**) Flux, Permeate concentration and NaCl rejection with CTAB + 3.5 wt.% NaCl, SDS + 3.5 wt.% NaCl and Tween 80 + 3.5 wt.% NaCl, respectively.

CTAB has the least hydrophilic–lipophilic balance (HLB) value, indicating higher hydrophobic–hydrophobic interactions with the membrane [7]. Also, CTAB is a positively charged surfactant, and the electrostatic interaction with the membrane surface leads to a loss of membrane hydrophobicity and the lowering of the surface tension [16]. The M_4S_ proved stable even at concentrations of 1.2 mM, even higher than its CMC of 0.9 mM, indicating the amphiphobic nature of the membrane. The membrane exhibited a stable flux of ≃17 LMH up to a CTAB concentration of 1.2 mM (240 min) (Figure 8a). Upon further addition of 0.3 mM of CTAB, the permeate flux increased by ≃47%. Concurrently, the permeate TOC concentration increased from 1.8 to 5.8 ppm at 300 min for 1.5 mM CTAB. An upsurge in the flux and subsequent decrease in permeate quality is attributed to the instantaneous membrane pore wetting. DI water wash (followed by drying) was effective in membrane regeneration, as indicated by the identical WCA obtained. The presence of salt in the feed could lower the CMC and further reduce the feed’s surface tension, leading to a slightly greater permeate water flux. Interestingly, the M_4S_ exhibited enhanced resistance to membrane wetting for up to a higher concentration (2.1 mM) of CTAB in the feed (Figure 8b).

It can be inferred that in the presence of salt, the adsorption of surfactants was hindered by concentration polarization due to the high concentration of NaCl. However, the migration of the wetting front caused steady pore wetting to progress. Hence the main factor affecting the membrane wetting was the transport of CTAB to the vapor–liquid interface rather than the adsorption rate as evidenced by other researchers [7].

SDS is an anionic surfactant with a higher HLB value (40), indicating a hydrophilic character, and the surfactant has more affinity to water than the hydrophobic membrane. The negative charge of the SDS electrostatically hinders the adsorption onto the membrane surface. The CENM membrane could withstand much higher concentrations, as high as 16 mM SDS (flux: 12 to 14 LMH and permeate concentration: <4 ppm) up to 480 min in the feed (Figure 8c). The adsorption of the SDS is energetically highly favorable due to the higher interactions between the hydrophobic tail of the surfactant and the hydrophobic membrane surface and hence, could be controlled by the rate of surfactant transport to the wetting front [43]. The presence of 3.5 wt.% NaCl destabilizes the repulsive forces and increases the wetting tendency of the membrane at lower SDS concentrations, as seen in the Figure 8d. In the presence of salt, the membrane could withstand only up to 12 mM (300 min). Thereby, it is evident that in the presence of SDS, the membrane exhibited a stable performance (flux: 12 to 14 LMH and permeate concentration: <4 ppm) up to 480 min (SDS concentration: 18 mM). SDS with a hydrophobic tail and hydrophilic head would have formed a hydrophilic layer on the membrane surface and hydrophilic channels throughout the membrane pores, enabling liquid transfer rather than vapor transfer. However, the CENM with re-entrant morphology and amphiphobic character prevented the instantaneous formation of hydrophilic channels [44].

Figure 8e,f shows the vapor transport flux and rejection performance of membrane M_4S_ with Tween-80 and Tween-80 + 3.5 wt.% NaCl in the feed solution. A rapid increase in the permeate flux and decline in permeate quality could be observed in both cases, indicating an instantaneous wetting of the membrane. Tween-80 almost instantaneously wet the membrane, which could not be regenerated by water wash, as reported in previous studies [7]. The CMC value is very low; ideally, the rate of transport of the Tween-80 to the wetting front would be much slower compared to SDS or CTAB, which was not true in this case. However, unlike CTAB (with the same HLB value), the wetting phenomenon with Tween-80 was rapid, indicating complete membrane wetting.

The solution surface tension or the contact angles could not clearly describe this wetting phenomenon (at CMC-Tween-80 had a higher surface tension than SDS or CTAB). The higher molecular weight of 1310 Da and the low HLB value of the polymeric Tween-80 could contribute to the membrane wetting [45]. The polymeric surfactant micelle could also be more prominent in size that adsorbs on the hydrophobic membrane, thereby shielding the hydrophobic domains, which leads to inevitable pore wetting. The wet membrane showed only 85.37% recovery of its WCA (120.81 ± 3.5°), indicating an irreversible wetting. The presence of NaCl further destabilized the membrane performance.

**Figure 9:**
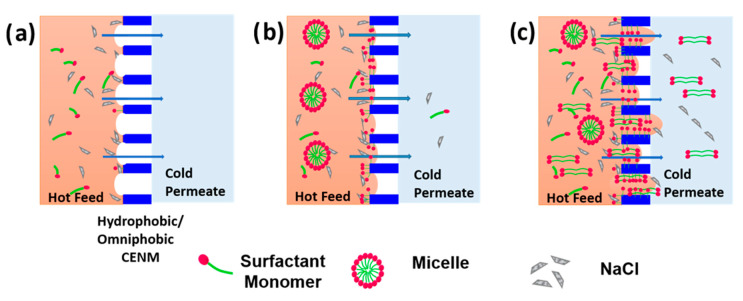
Illustration of different phases of membrane wetting by surfactant containing saline wastewater. (**a**) non-wetting phase, (**b**) concentration-dependent phase and (**c**) concentration-independent phase.

For charged surfactants, the membrane wetting can be divided into three phases—(a) non-wetting phase, (b) concentration-dependent phase and (c) concentration-independent phase (Figure 9). In the non-wetted phase, the surfactant concentration is generally low and often below the CMC (or approaching), where the surfactants exist as monomers (in a free state) in the solution. This phase causes no loss of permeate quantity or quality. As the concentration of the surfactants gradually increases, the surfactants form a more stable form (micelles) in the bulk feed solution. The surface tension of the solution continuously decreases until the CMC is reached, after which it is quite stable. In the concentration-dependent phase, the surfactant monomers adsorb onto the membrane surface (hydrophobic or electrostatic interactions), owing to their intrinsic properties (size, charge or HLB value). Finally, an equilibrium is attained between the surfactant transported from bulk to the vapor/liquid interphase and the adsorption on the membrane surface. Also, the increased adsorption density decreases the mixing energy (Gibbs free energy) at the membrane–feed interface. However, the adsorbed surfactant monomers construct hydrophilic channels that facilitate the intrusion of water molecules through the membrane’s pores. In the concentration-independent phase, the degree of wetting reaches a plateau indicating a complete membrane wetting. The surfactants exhibit an autophilic effect and absorb ahead of the wetting front, making the pore hydrophilic. The wetting progresses through capillary action leading to the formation of hydrophilic flow paths [46]. The high concentration of salts in the feed alters the membrane wetting as they increase the H-bonding of water, reducing surface tension and pH [6]. In this study, the presence of salt diminished the M_4S_ performance for the anionic SDS feed solution. However, the onset of wetting was delayed (stable for higher concentrations) for the cationic CTAB feed solution. The membrane wetting by the non-ionic Tween-80 surfactant was instantaneous in the presence of salt [47].

A comparison of this study with the state-of-the-art literature is given in Table 3. The CENMs prepared in this study hold good potential for water recovery from challenging wastewater and must be explored further to elucidate the mechanisms of membrane wetting in long-term applications.

## 4. Conclusions

Coaxial electrospinning is a facile technology to fabricate nanofibrous membranes with 3D hierarchical/multiscale roughness. The synergistic effect of Al_2_O_3_ NPs and FAS facilitated the formation of re-entrant morphology and enhanced membrane hydrophobicity/oleophobicity. The optimum membrane exhibited contact angles in the order WCA (142.5 ± 0.9°) > ethanol CA (121.6 ± 2.6°) > hexadecane (119.6 ± 1.8°) > 2.4 mM CTAB (92.54 ± 2.4°). The membrane proved stable enough to recover water from 7 wt.% and 10.5 wt.% without loss in permeate quality. The M_4S_ demonstrated a consistent vapor transport flux of 8–9 LMH for every cycle, and the salt rejection never fell below 99.97%. The vapor transport flux decreased with the increase in feed solution concentration, as expected, and almost complete flux recovery was obtained for over five cycles of operation (60 h). The role of various types of surfactants, such as CTAB (cationic), SDS (anionic) and Tween-80 (non-ionic), on membrane wetting was investigated in detail. CTAB, with a low HLB value, has a high affinity (electrostatic) to the negatively charged membrane. At concentrations greater than CMC, these surfactants adsorb at the surface of the membrane/pore, forming hydrophilic channels and leading to progressive wetting of the membrane. The presence of salts hinders the adsorption of the CTAB, leading to a more excellent resistance to membrane wetting up to 2.1 mM concentration in the feed (compared to 1.5 mM without salt). The M_4S_ displayed outstanding resistance to wetting by SDS (high HLB value) up to 16 mM concentration and further addition of SDS lead to a loss of permeate quality. Here the hydrophobic–hydrophobic interactions lead to the adsorption of SDS on the membrane. The addition of salts blocks the membrane surface charge leading to rapid adsorption of the surfactants at lower concentrations causing membrane wetting (12 mM as compared to 16 mM without salt). The CENM displayed the least resistance to membrane wetting when Tween-80 was present in the feed. In the presence of salt, instantaneous membrane wetting occurred with a loss of permeate quality. Overall, the CENMs hold good potential for water recovery from hypersaline and surfactant-containing wastewater and must be further explored for long-term performance.

## Figures and Tables

**Table 1:** Composition of the CENMs.

Membrane Code	Core Solution	Sheath Solution		FAS	Type of FAS Modification
		Polymer Solution	Alumina		
M_0_	15% PVDF-HFP + 85% Solvent Mixture (DMF: Acetone: 7:3)	9% PVDF-HFP + 85% Solvent Mixture (DMF: Acetone: 7:3)	0.1%	0%	No
M_1C_			0.1%	0.025%	Surface Coating
M_2S_			0.1%	0.025%	In Sheath solution
M_3S_			0.2%	0.025%	In Sheath solution
M_4S_			0.2%	0.05%	In Sheath solution

**Table 2:** Composition of feed solution.

Feed Solution	Concentration	Properties
Synthetic Hypersaline solutions	7 wt.% NaCl	Conductivity ≃ 98,450 µS/cm
	10.5 wt.% NaCl	Conductivity ≃ 98,450 µS/cm
Synthetic Surfactant wastewater	CTAB Initial CTAB concentration: 0.3 mM	Conductivity ≃ 54,690 µS/cm Total Organic Carbon: 152.6 ppm
	CTAB + 3.5 wt.% NaClInitial CTAB Concentration: 0.3 mM	
	SDS (Initial SDS Concentration: 2 mM)	Conductivity ≃ 54,690 µS/cm Total Organic Carbon:198.65 ppm
	SDS + 3.5 wt.% NaCl (Initial SDS Concentration: 2 mM)	
	Tween-80 (Initial Concentration: 0.106 mM)	Conductivity ≃ 54,690 µS/cm Total Organic Carbon: 164.5 ppm
	Tween-80 + 3.5 wt.% NaCl (Initial Concentration: 0.106 mM)	

**Table 3:** State-of-the-art comparison of membrane anti-wetting stability using surfactants.

Surfactant Used	Feed Solution Concentration	Membrane Used	Performance of the Membrane	Reference
CTAB, SDS, Tween 20	50 mg/L of surfactant + 35 mg/L of NaCl	Commercial hydrophobic PVDF	Wetting time for (i) CTAB ≃ 20 min (ii) SDS ≃ 50 min (iii) Tween 20 ≃ 37 min CTAB and SDS achieved a good recovery, and the wetting time only reduced by 17.4% and 10.6%, respectively. Poor recovery was obtained for the membranes wetted by Tween 20.	[7]
CTAB, SDS, Tween 20, 2-EHS, SDBS (0.5 × CMC) (With and without NaCl)	(0.5 × CMC) + 35 g/L NaCl	Flat-sheet PVDF commercial membrane	Wetting time and flux decline (i) CTAB ≃ 1500 min and 5% (ii) CTAB + 3.5% NaCl ≃ 1500 min and 11% (iii) Tween 20 ≃ 1500 min and 4% (iv) Tween 20 + 3.5% NaCl ≃ 1500 min and 20% (v) SDS ≃ 1500 min and 8% (vi) SDS + 3.5% NaCl ≃ 1200 min and 22%	[6]
SLS, SDS, CTAB, DTAB	SDS, SLS—0.0003 mM + 3.5% NaCl DTAB—0.0005 mM + 3.5% NaCl CTAB—0.0001 mM + 3.5% NaCl	PVDF membrane coated with fluorinated Silver nanoparticles	(i) CTAB Study time—3 h (100% decline in normalized flux) and salt rejection remained > 99.9%) (ii) SDS Study time -8 h (50% decline in normalized flux) and salt rejection reduced to 99.84%) (iii) SLS Study time—8 h(75% decline in normalized flux) and salt rejection 100%) (iv) Study time—12 h (Normalized flux remained ≃ 1 and salt rejection 100%)	[44]
CTAB, SDS, Tween 80	10–50 mg/L + 0.5 g/L of gasoline + 30 g/L of NaCl	PDADMAC/PAA semi-IPN hydrogel-coated PVDF membrane	(i) SDS Study time—480 min; Concentration—50 mg/L (Flux—4 to 5 Kg/m^2^.h; Conductivity—20 to 30 µS/cm) (ii) CTAB Study time—480 min; Concentration—30 mg/L (Flux—4 to 5 Kg/m^2^.h; Conductivity—20 to 30 µS/cm) (iii) Tween 80 Study time—480 min; Concentration—10 mg/L (Flux—4 to 5 Kg/m^2^.h; Conductivity—20 to 40 µS/cm)	[48]
CTAB, SDS, Tween 20 (With and Without 3.5% NaCl)	Increasing surfactant concentration with time	Alumina incorporated coaxially electrospun PVDF-HFP amphiphobic membrane	Membrane wetting time and feed concentration (i) CTAB—260 min and 1.5 mM (ii) CTAB + 3.5% NaCl—400 min and 2.4 Mm (iii) SDS—480 min and 16 mM (iv) SDS + 3.5% NaCl—300 min and 12 mM (v) Tween 80—120 min and 0.16 mM (vi) Tween 80 + 3.5% NaCl—60 min and 0.10 mM	This Study

## Supplementary Materials

The following supporting information can be downloaded at: https://www.mdpi.com/article/10.3390/polym14245350/s1, Section S1: Characterization of nanoparticles and membranes, Table S1: Properties of the different surfactants, Table S2: Elemental Composition of Membrane M4S, Table S3: Contact angle of M4S with different feed solutions and surface tension of different feed solutions on the membrane surface (M4S).
