# Supplementary material for: Coaxial Electrospun Nanofibrous Membranes for Enhanced Water Recovery by Direct Contact Membrane Distillation
Source: Polymers (Basel). 2022 Dec 7;14(24):5350. doi: 10.3390/polym14245350 (PMC9784477; doi:10.3390/polym14245350)
Supplement: Supplementary file 1 [file polymers-14-05350-s001.zip › polymers-2037288-supplementary.pdf]

Coaxial Electrospun Nanofibrous Membranes for Enhanced Water Recovery by Direct Contact Membrane Distillation

Vivekanandan Sangeetha[a], Noel Jacob Kaleekkal[a*], Saravanamuthu Vigneswaran[b,c*]

[a] *Membrane Separation Group, Department of Chemical Engineering, National Institute of Technology Calicut, Kozhikode, Kerala – 673601, India.*

[b] *Centre for Technology in Water and Wastewater, School of Civil and Environmental Engineering, University of Technology Sydney, Sydney, NSW 2007, Australia.*

[c] *Faculty of Sciences & Technology (RealTek), Norwegian University of Life Sciences, P.O. Box 5003, NO-1432 Ås, Norway*

E-mail: Saravanamuth.Vigneswaran@uts.edu.au

Table S1. Properties of the different surfactants

Surfactant	Molecular Formula	Chemical Structure	Molecular Weight, g.mol^{-1}	Surfactant Charge	Hydrophilic-Lipophilic Balance (HLB) Value	Critical Micelle Concentration (CMC) value	Reference
Cetyl Triammonium Bromide (CTAB)	$C_{19}H_{42}NBr$	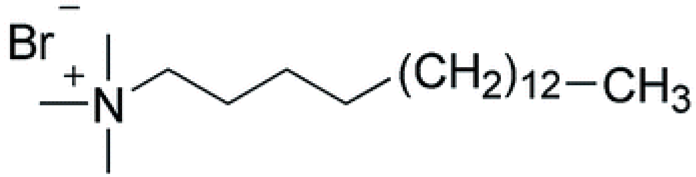	364.46	Cationic	15	0.9 mM	[6, 44]
Sodium Dodecyl Sulphate (SDS)	$C_{12}H_{25}SO_4Na$	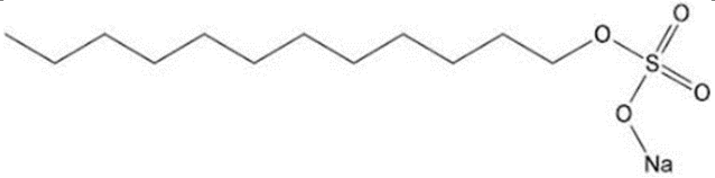	288.38	Anionic	40	8 mM	[6]
Tween - 80	$C_{64}H_{124}O_{26}$	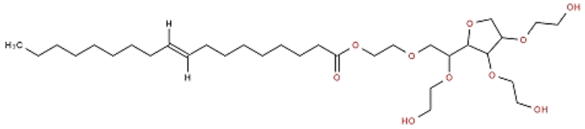	1310	Non-ionic	15	0.106 mM	[45]

Table S2. Elemental Composition of Membrane M4S

Element	Line Type	Apparent Concentration	k Ratio	Wt%	Wt% Sigma	Atomic %	Standard Label
C	K series	3.73	0.03725	37.95	0.44	49.11	C Vit
O	K series	0.22	0.00073	0.85	0.19	0.82	SiO2
F	K series	33.86	0.06649	61.18	0.45	50.06	CaF2
Al	K series	0.00	0.00002	0.02	0.10	0.01	Al2O3
Total:				100.00		100.00	

Table S3. Contact angle of M4S with different feed solutions and surface tension of different feed solutions on the membrane surface (M_{4S})

Sl. No	Sample	Solution Contact Angle (initial)	Solution contact angle (t = 10 min)	Solution Surface Tension (mN/m)
1	DI Water			71.82
2	0.6 mM CTAB	135.40	132.20	34.81
3	0.9 mM CTAB	129.80	123.6	32.17
4	1.8 mM CTAB	123.20	117.30	33.19
5	0.6 mM CTAB + 3.5 wt. % NaCl	122.20	118.40	33.40
6	0.9 mM CTAB + 3.5 wt. % NaCl	119.40	115.20	32.01
7	1.8 mM CTAB + 3.5 wt. % NaCl	119.20	102.40	33.94
8	4 mM SDS	123.00	120.00	36.11
9	8 mM SDS	120.8	118.20	34.99
10	16 mM SDS	The drop could not be stabilized to drop on the membrane surface		32.40
11	4 mM SDS + 3.5 wt. % NaCl			32.57

12	8 mM SDS + 3.5 wt. % NaCl			30.29
13	16 mM SDS + 3.5 wt. % NaCl			30.42
14	0.053 mM Tween 80	127.6	125.40	39.66
15	0.106 mM Tween 80	133.5	130.4	40.20
16	0.053 mM Tween 80 + 3.5 wt. % NaCl	125.30	119.20	41.62
17	0.106 mM Tween 80 + 3.5 wt. % NaCl	127.6	124.90	40.08
